# Cytosolic localization and *in vitro* assembly of human *de novo* thymidylate synthesis complex

**DOI:** 10.1111/febs.16248

**Published:** 2021-11-12

**Authors:** Sharon Spizzichino, Dalila Boi, Giovanna Boumis, Roberta Lucchi, Francesca Romana Liberati, Davide Capelli, Roberta Montanari, Giorgio Pochetti, Roberta Piacentini, Giacomo Parisi, Alessio Paone, Serena Rinaldo, Roberto Contestabile, Angela Tramonti, Alessandro Paiardini, Giorgio Giardina, Francesca Cutruzzolà

**Affiliations:** ^1^ Department of Biochemical Sciences Sapienza University of Rome Rome Italy; ^2^ Istituto di Cristallografia Consiglio Nazionale delle Ricerche Rome Italy; ^3^ Center for Life Nano & Neuro‐Science Fondazione Istituto Italiano di Tecnologia (IIT) Rome Italy; ^4^ Istituto di Biologia e Patologia Molecolari Consiglio Nazionale delle Ricerche Rome Italy; ^5^ Laboratory affiliated to Istituto Pasteur Italia – Fondazione Cenci Bolognetti Rome Italy; ^6^ Present address: Grup d'Enginyeria de Materials (GEMAT) Institut Químic de Sarrià (IQS) Universitat Ramon Llull (URL) Barcelona Spain

**Keywords:** cancer metabolism, protein–protein complex, purine synthesis, thymidylate synthesis, transient interactions

## Abstract

*De novo* thymidylate synthesis is a crucial pathway for normal and cancer cells. Deoxythymidine monophosphate (dTMP) is synthesized by the combined action of three enzymes: serine hydroxymethyltransferase (SHMT1), dihydrofolate reductase (DHFR) and thymidylate synthase (TYMS), with the latter two being targets of widely used chemotherapeutics such as antifolates and 5‐fluorouracil. These proteins translocate to the nucleus after SUMOylation and are suggested to assemble in this compartment into the thymidylate synthesis complex. We report the intracellular dynamics of the complex in cancer cells by an *in situ* proximity ligation assay, showing that it is also detected in the cytoplasm. This result indicates that the role of the thymidylate synthesis complex assembly may go beyond dTMP synthesis. We have successfully assembled the dTMP synthesis complex *in vitro*, employing tetrameric SHMT1 and a bifunctional chimeric enzyme comprising human thymidylate synthase and dihydrofolate reductase. We show that the SHMT1 tetrameric state is required for efficient complex assembly, indicating that this aggregation state is evolutionarily selected in eukaryotes to optimize protein–protein interactions. Lastly, our results regarding the activity of the complete thymidylate cycle *in vitro* may provide a useful tool with respect to developing drugs targeting the entire complex instead of the individual components.

## Graphical abstract

*De novo* thymidylate synthesis is a crucial pathway for cancer cells that rely on deoxythymidine monophosphate (dTMP) to fuel DNA synthesis. dTMP is synthesized by three enzymes forming a transient protein–protein complex. We report the intracellular dynamics and localization of the dTMP synthesis complex in cancer cells, as well as its successful assembly *in vitro*. Our results provide a useful tool to develop drugs targeting the entire complex instead of the individual components.

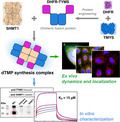

**Abbreviations**
5‐FU: 5‐fluorouracil
BLI: bio‐layer interferometry
CH_2_‐THF: 5,10‐methylene‐tetrahydrofolate
CHO‐THF: 5‐formyl‐tetrahydrofolate
BLI: bio‐layer interferometry
DAPI: 4′,6‐diamidino‐2‐phenylindole
DSF: differential scanning fluorimetry
DHF: dihydrofolate
DHFR: dihydrofolate reductase
dTMP: deoxythymidine monophosphate
dTMP‐SC: thymidylate synthesis complex
dUMP: deoxyuridine monophosphate
FWB: far western blotting
IF: immunofluorescence
IMAC: immobilized metal affinity chromatography
IP: immunoprecipitation
IPTG: isopropyl thio‐β‐d‐galactoside
is‐ PLA: *in situ* proximity ligation assay
o.n.: overnight
PBS: phosphate buffered saline
PDB: Protein Data Bank
PPI: protein–protein interactions
PVDF: poly(vinylidene difluoride)
SEC: size exclusion chromatography
SHMT: serine hydroxymethyltransferase
SPR: surface plasmon resonance
THF: tetrahydrofolate
*T*_m_
: melting temperature
TYMS: thymidylate synthase

## Introduction

Maintenance of physiological dNTP levels is critical for genome stability and any alteration in their levels has complex consequences [1]. Among the *de novo* nucleotide synthesis pathways, deoxythymidine monophosphate (dTMP) synthesis is active in many tissues and is critical for the proliferation of different tumours [2]; it also connects dNTP synthesis with folate and one‐carbon metabolism, a complex network of reactions controlling the synthesis of a number of different precursors and providing methylation and antioxidant power. All of the enzymes involved are strictly regulated at the transcriptional and translational level [3, 4, 5].

**Scheme 1:**
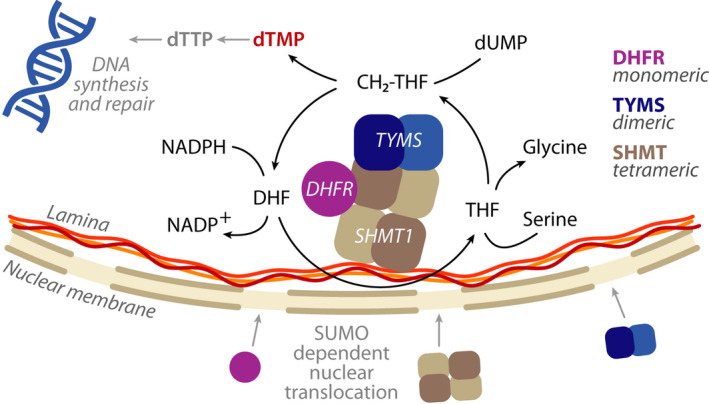
Scheme of the nuclear dTMP‐SC catalytic cycle. SHMT1, DHFR and TYMS are SUMOylated and translocate to the nucleus during G1/S‐phase, where they are proposed to assemble to form the dTMP synthesis complex (dTMP‐SC), anchored to the nuclear lamina [7]. The oligomeric state of the three enzymes is also reported.

dTMP is synthesized starting from deoxyuridine monophosphate (dUMP) by thymidylate synthase (TYMS) (EC:2.1.1.45) in synergy with two other folate‐dependent enzymes of the folate cycle: serine hydroxymethyltransferase (SHMT1) (EC:2.1.2.1) and dihydrofolate reductase (DHFR) (EC:1.5.1.3) [5]. SHMT1 produces 5,10‐methylenetetrahydrofolate (CH_2_‐THF) from tetrahydrofolate (THF) using serine as one‐carbon source. The carbon atom is then transferred from CH_2_‐THF to dUMP by TYMS to form dTMP and dihydrofolate (DHF); finally, the NADPH‐dependent reduction of DHF to THF, catalysed by DHFR, closes the thymidylate synthesis cycle (Scheme 1). dTMP synthesis appears to be compartmentalized to the mitochondria [6] and the nucleus, where it sustains DNA replication during S‐phase or after DNA damage. The three enzymes undergo a SUMO‐dependent translocation into the nucleus, where they were shown not only to assemble in the thymidylate synthesis complex (dTMP‐SC), anchored to the lamina by SHMT1, but also to be present at DNA synthesis sites and interact with the DNA replication machinery [7, 8, 9, 10] (Scheme 1). Complex formation in the nucleus is assumed to be responsible for dTMP synthesis and to prevent genome uracil misincorporation [7, 11, 12].

**Fig. 1:**
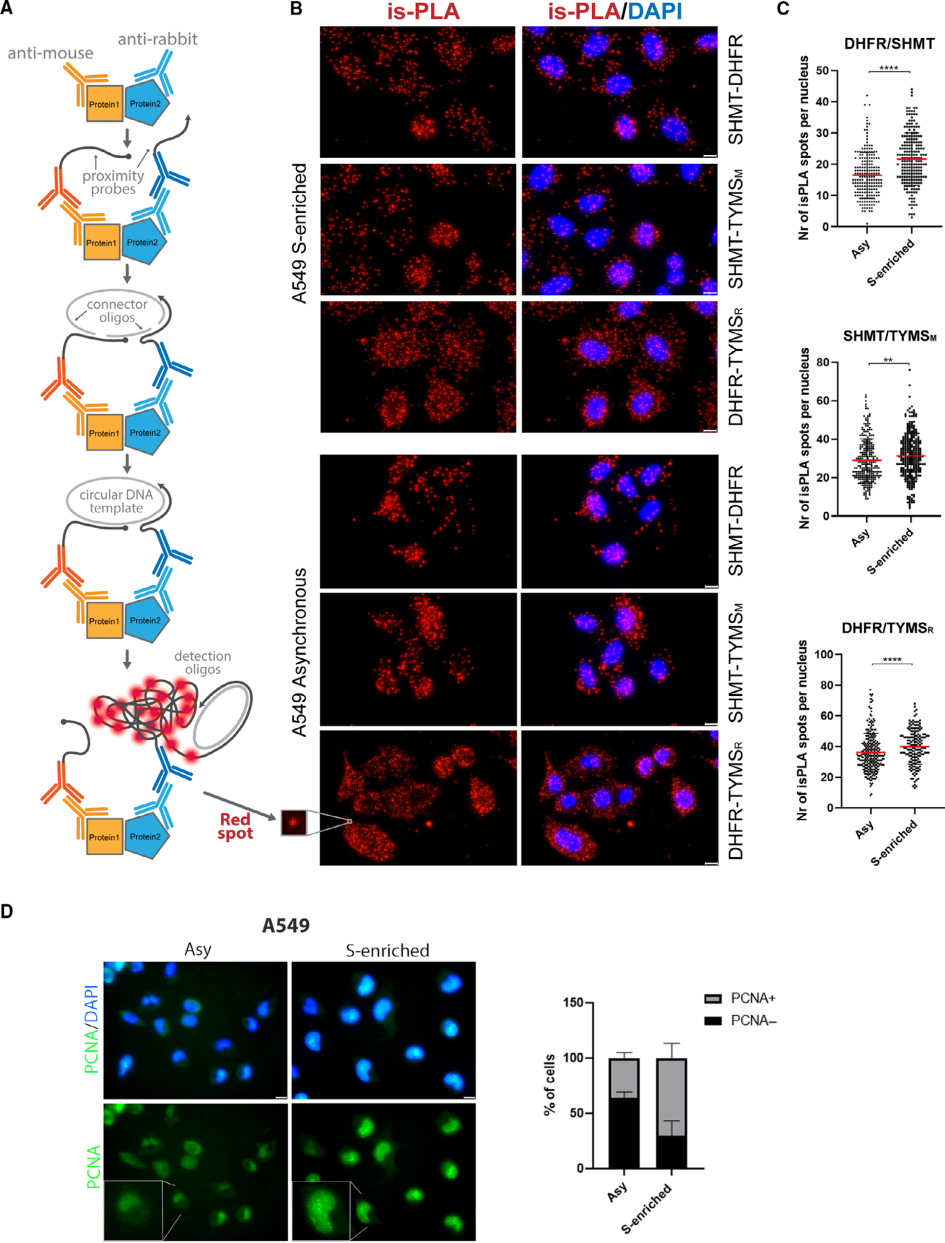
Identification of proteins proximity in cancer cell lines. (A) Scheme of the is‐PLA: 1, primary antibodies bind specific proteins; 2, secondary antibodies conjugated with oligonucleotides (proximity probes) bind to anti‐rabbit or mouse primary antibodies; 3, if the two proteins are interacting (< 40 nm apart), the proximity probes can hybridize with the two connector oligos; 4, the ligation step produces a circular DNA template; 5, circular DNA is then amplified by DNA polymerase. Detection oligos coupled to fluorochromes hybridize to repeating sequences in the amplicons yielding the is‐PLA signal detected by fluorescent microscopy as discrete spots (inset). (B) is‐PLA signals, corresponding to DHFR/SHMT, SHMT/TYMS and DHFR/TYMS interactions, are shown (red dots). TYMS_M_ and TYMS_R_ both indicate antibodies against TYMS but anti‐mouse and anti‐rabbit, respectively. The is‐PLA spots of interaction of proteins are shown in A549 cells synchronised in the S‐phase after a 24‐h single thymidine block (upper) or in A549 asynchronous cells (bottom). Scale bars = 10 µm. The merge with DAPI signal is shown on the right. Controls are shown in Fig. 4B–F. (C) Nuclear localization of the is‐PLA signal (number of spots within the normalized nucleus area). In all of the experiments, the number of PLA spots increase in the S‐enriched cells (*t*‐test: **P < 0.005; ****P < 0.0001). At least 160 cells were analysed per condition. (D) Left: representation of PCNA positive cells, corresponding to S‐phase of the cell cycle. Scale bar = 10 µm. Right: immunofluorescence signal in an asynchronous population or after a single thymidine block of A549. The histogram on the right shows the percentage of PCNA positive or negative cells after and before synchronisation. At least 500 cells were counted per conditions, from three independent experiments; SD values are shown. After synchronisation, the cells in S‐phase comprise approximately 80% of the cellular population.

Given their role in cell proliferation, two out of three of the dTMP‐SC enzymes (i.e. TYMS and DHFR) are targets of widely used chemotherapeutic drugs such as 5‐fluorouracil (5‐FU) and antifolates such as methotrexate or pemetrexed [13, 14, 15]. In the cells, 5‐FU is converted to fluorodeoxyuridine‐monophosphate, which covalently binds to TYMS acting as a suicide inhibitor, whereas the antifolates are cofactor analogues acting as competitive inhibitors [2]. Given the importance of the targeted enzymes in nucleobases metabolism, the side effects of these treatments are severe [16]. Moreover, despite these drugs being in use from the early 1960s onward, the major drawback of this chemotherapy is that cancer cells can rewire their metabolism in response to the lack of THF and dTMP by increasing the expression of both TYMS and DHFR or by upregulating ATP‐driven efflux transporters [17, 18, 19]. To overcome these issues, a great effort has been made to find new inhibitors targeting other enzymes, such as SHMT [20, 21, 22, 23, 24, 25], although with little results to date.

A fascinating alternative strategy would be to target protein–protein interactions (PPI) instead of the single enzymes. Protein complexes and PPI are commonly formed by cells to increase the efficiency, tunability and control over crucial metabolic pathways [26]. Many of these protein assemblies undergo complex dynamics, often controlled by other cellular components such as nucleic acids and lipids and even segregate in membrane‐less intracellular compartments [27, 28]. Interfering with PPI to highjack protein metabolism is a challenging goal that is feasible only when biochemical and structural data of the target PPI become available [29]. The interaction between the three enzymes of the dTMP‐SC has been demonstrated only by immunoprecipitation (IP) and was suggested to take place only in the nucleus [7, 30]. Several features of the complex are still unclear, including how the three enzymes come together, and whether the complex is needed to provide dTMP *in situ* during DNA replication/repair or to enhance the catalytic efficiency through a substrate tunnelling mechanism or to generate a binding region to anchor the complex to DNA. Nor it is clear whether the dTMP‐SC is formed in the cytosol and needed to control dTTP local pools or whether it may be involved in other functions.

In the present study, we aimed to understand how the dTMP‐SC coherently assembles and functions, ultimately providing the molecular basis regarding how it orchestrates dTMP metabolism.

Our results show that the dTMP‐SC is abundant in the cytoplasm of both S‐phase synchronised and non‐synchronised lung cancer cells, suggesting that the interaction between these enzymes may go beyond the nuclear dTMP synthesis and embrace novel regulatory pathways yet to be unveiled. We have successfully assembled the dTMP synthesis complex *in vitro*, employing tetrameric SHMT1 and a bifunctional chimeric enzyme comprising human TYMS and DHFR, in which the two enzymes were still active. To the best of our knowledge, the dTMP‐SC has never been isolated before. Only one study has reported the interaction of human TYMS and DHFR, which were shown to form a very faint complex *in vitro* [31]. Lastly, we show here that the SHMT1 tetramer is required for optimal complex assembly, suggesting that this aggregation state is evolutionary selected in eukaryotes to optimize PPI, which may indeed represent a promising drug target.

## Results

### Detection of the dTMP‐SC complex in the cytosol and nucleus of lung adenocarcinoma A549 and HeLa cells by an *in situ* proximity ligation assay

To monitor the formation of the dTMP‐SC and to obtain novel information on its localization in space and time, we performed an *in situ* proximity ligation assay (is‐PLA). A major benefit of PLA is that it does not require modification or tagging of the proteins of interest, allowing endogenous interactions to be detected with greater sensitivity with respect to co‐immunopurification methods previously employed for the dTMP‐SC [7]. We used both non‐synchronised and S‐phase synchronised lung adenocarcinoma cancer cells (A549) to determine the interactions between all the possible couples in the ternary complex: SHMT1–TYMS–DHFR. Is‐PLA uses ordinary primary antibodies to detect the proteins of interest, which are then revealed using secondary antibodies conjugated to DNA oligonucleotides, producing a fluorescence signal in the form of a spot, given by the ligation and amplification of the oligonucleotides that occurs only when the two proteins are interacting or closer than 40 nm (Fig. 1A) [32]. As shown in Fig. 1B, we could detect the positive PLA signals for all the combinations of the three proteins in both synchronous and asynchronous A549 cells. Interestingly, the complex was more abundant in the cytoplasm than in the nucleus, and nuclear localization appears to be enriched in synchronised S‐phase cells only to some extent (Fig. 1C,D).

**Fig. 2:**
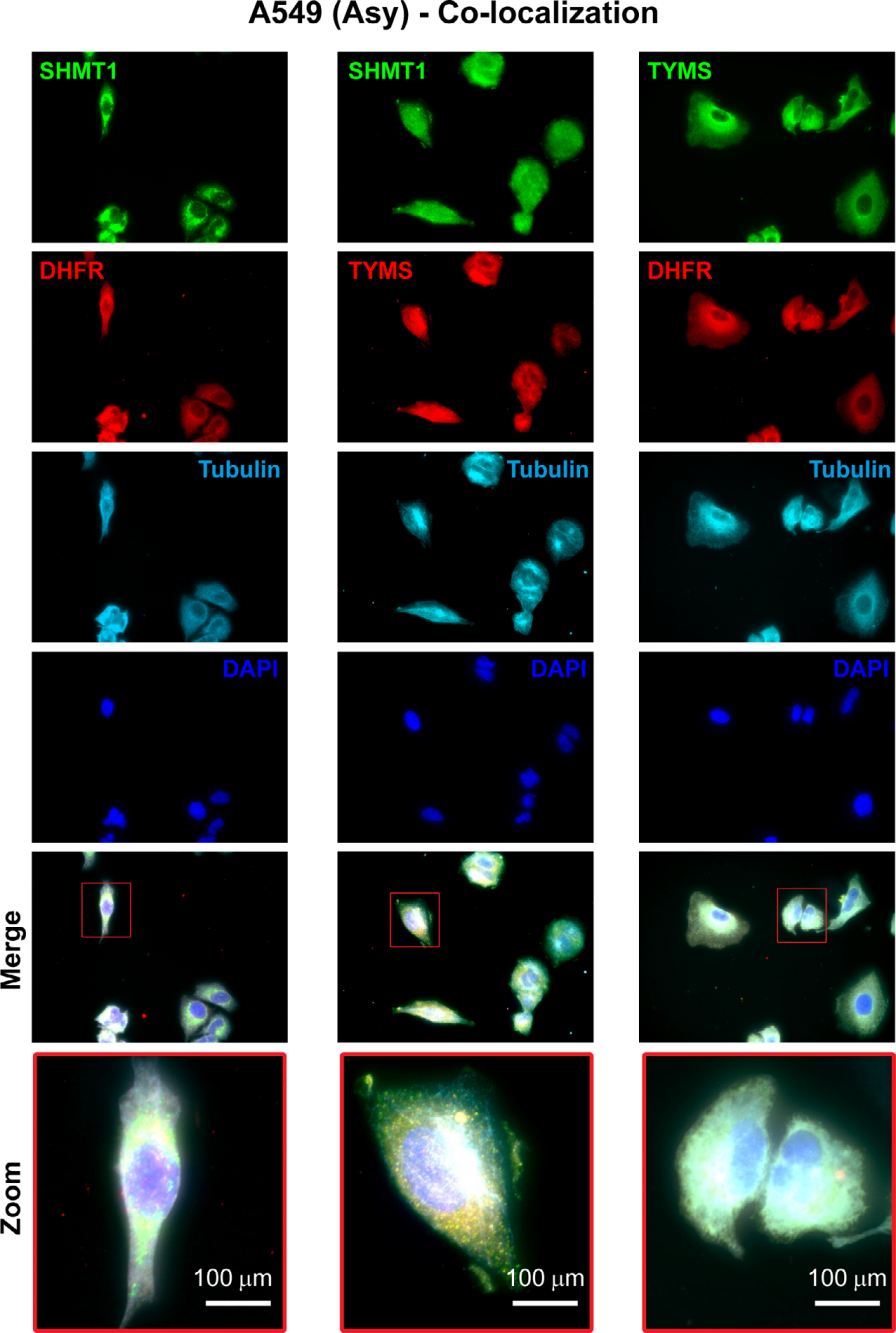
Immunofluorescence co‐localization analysis. (A) Co‐localization of DHFR/SHMT1, SHMT1/TYMS and TYMS/DHFR in asynchronous A549 cells. Co‐localization is evident both in the cytosol (tubulin alpha, cyan) and in the nucleus (DAPI, blue) for all the three couples. The original red fluorescence signal of tubulin was changed to cyan for better visualization of the merged images. The merge of green, red and cyan pixels yields white pixels.

**Fig. 3:**
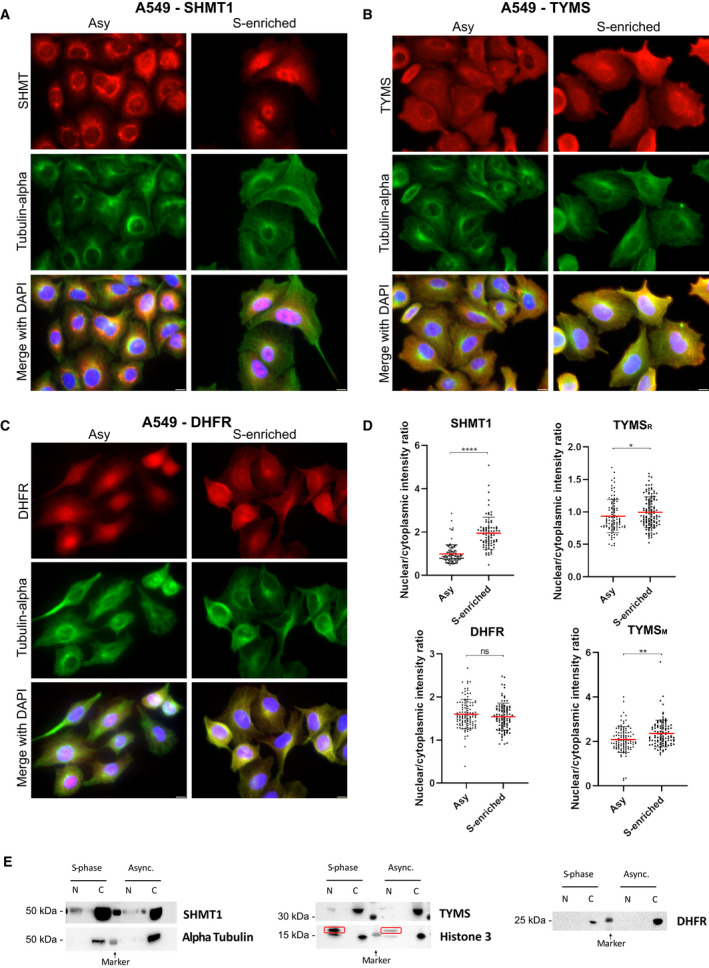
Immunofluorescence analysis. (A–C) Compartmentalization of the three proteins with respect to the cellular phase by Immunofluorescence analysis. SHMT1 (A), DHFR (B) and TYMS (C) show a cytoplasmic localization both in the asynchronous and S‐phase synchronised cells. Scale bar = 10 µm. (D) Nuclear localization (as deduced by the normalized intensity of the fluorescence signal) is clearly more abundant in the S‐enriched cells for SHMT1; a slight increase is observed for TYMS (with both the antibodies used in the PLA experiments; rabbit, TYMS_R_; and mouse, TYMS_M_; see Materials and methods). No change was observed for DHFR. (*t*‐test: *P < 0.05; **P < 0.005; ****P < 0.0001). Error bars represent the SD. At least 80 cells per condition from two independent experiment were analysed. (E) Western blot of subcellular fractionation of both asynchronous and S‐phase enriched A549 cell lines. Western blotting analysis was performed using a 1 : 1000 dilution of the primary antibodies. For Histone H3, the correct band is boxed in red. The other bands detected in the cytosol have a molecular weight lower than 15 kDa and are also present in the nuclear fractions. Giving that the molecular weight of Histone H3 is 17 kDa, and the bands recur in all the four lanes, it is plausible that they are detected because of non‐specific interactions of the primary or secondary antibody. In this experiment, the quantitative analysis was not performed, but it is still possible to detect the bands of SHMT1 and TYMS only in the nucleus of the S‐phase enriched cells. As shown in the IF experiments, it was not possible to detect an increase of the presence of DHFR in the nucleus of the synchronised cells.

**Fig. 4:**
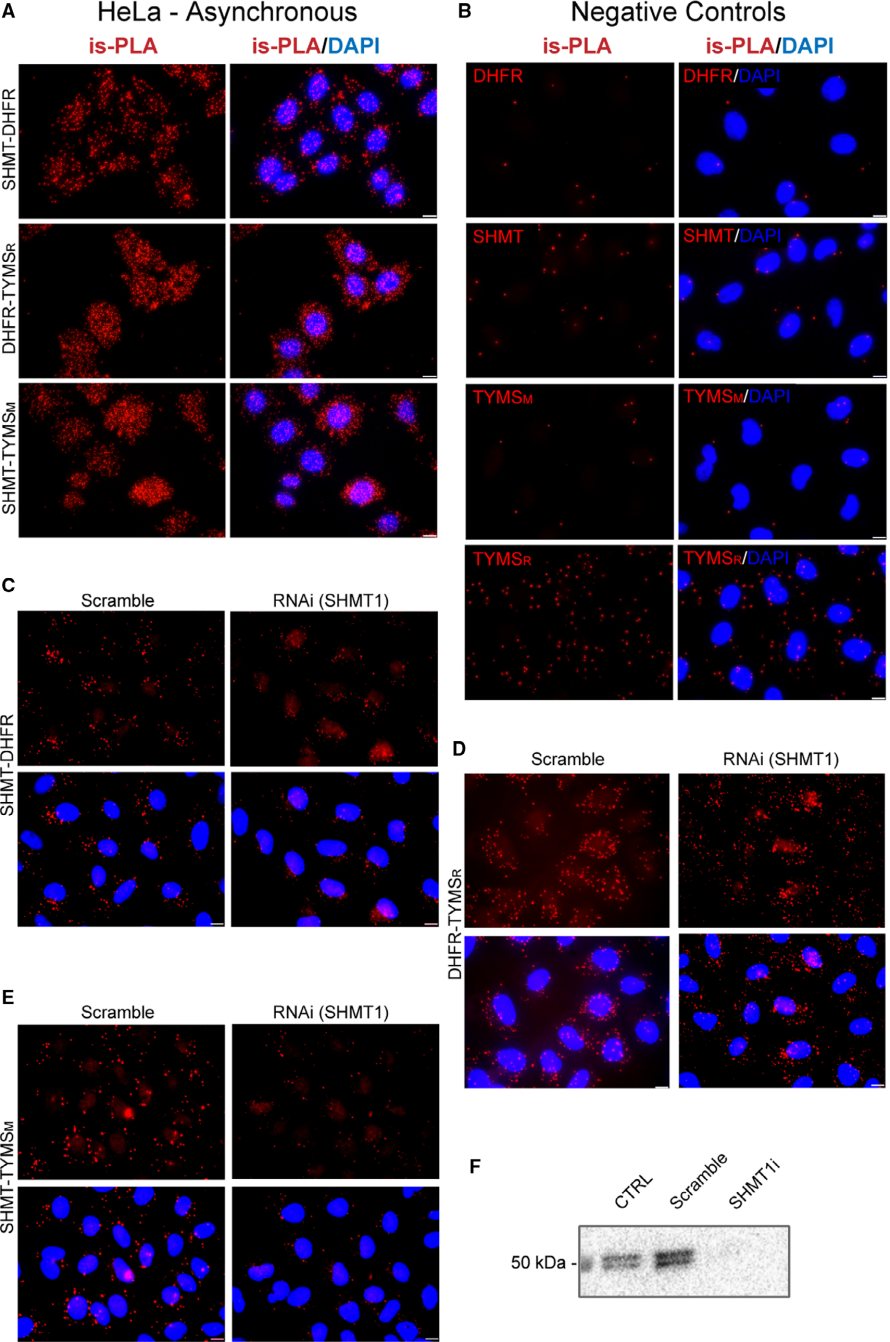
is‐PLA analysis on HeLa cells and is‐PLA negative controls on A549 cells. (A) PLA signal of protein interaction is shown in HeLa non‐synchronized cells. (B) Negative controls of is‐PLA signals. PLA experiment was performed without one of the primary antibodies (A549 cells). In these conditions, there is no or little PLA signal. TYMS_M_ and TYMS_R_ both indicate antibodies against TYMS but anti‐mouse and anti‐rabbit, respectively. (C–E) is‐PLA for the three protein–protein interaction performed on A549 cells 48 h after transfection with scrambled or siRNA for *shmt1*. For the DHFR/SHMT1 and TYMS/SHMT1 interactions the PLA signal is significantly lower in the RNAi cells, whereas, for the DHFR/TYMS interaction, the signal of the scramble and RNAi samples is similar. For all panels (A‐E), the scale bar in the bottom right corner is 10 µm. (F) SHMT1 expression control of RNAi.

To confirm the latter observation, we performed a co‐localization analysis (Fig. 2) and independently detected the presence of the single proteins (SHMT1, TYMS and DHFR) in the cytoplasm and in the nucleus of both synchronous and asynchronous A549 cells by immunofluorescence (IF) (Fig. 3A–D) and by western blotting after cell fractioning (Fig. 3E). The three proteins were always present in the cytoplasm, whereas nuclear localization was more abundant in S‐enriched cells only for TYMS and SHMT1 (Fig. 3D). This was particularly evident for the latter, suggesting that nuclear translocation of SHMT1 may drive the increase of dTMP‐SC formation in the nucleus during the S‐phase. Overall, the PLA results suggest that, under these conditions, the dTMP‐SC is located in the cytoplasm, as well as in the nucleus. This distribution was further confirmed in asynchronous HeLa cells (Fig. 4A). No PLA signal was observed when using only one antibody (Fig. 4B) or when the levels of SHMT1 were lowered by RNA interference, indicating that observed signal is specific (Fig. 4C–F).

These results indicate that the three enzymes are able to assemble the dTMP‐SC complex even in the absence of DNA or lamina proteins as binding partners [7], suggesting that it is possible to assemble the dTMP‐SC *in vitro* starting from the purified proteins. Our strategy to achieve this goal was to obtain a binary interaction, by assembling SHMT1 with a TYMS‐DHFR fusion protein.

### Rational design of a chimeric bifunctional protein encompassing DHFR and TYMS

**Fig. 5:**
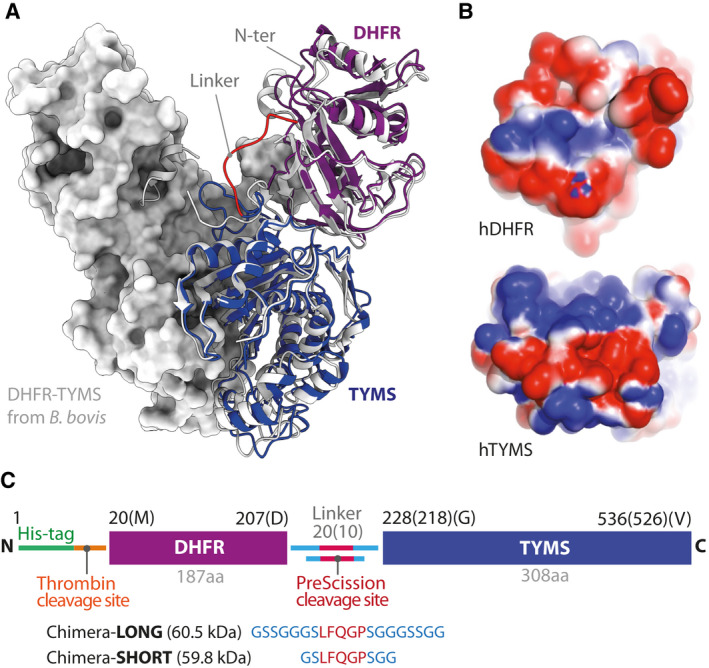
Designing human DHFR‐TYMS Chimera. (A) Cartoon representation of the chimeric model of human DHFR‐TYMS (in purple and blue, respectively; the linker is shown in red; the N‐terminus position is also indicated) superposed with the structure of the bifunctional enzyme from *B. bovis* (PDB ID: 3I3R [35]) in light grey. The enzyme is dimeric, the partner subunit is shown as surface representation. (B) Electrostatic surface potentials, as calculated using apbs (Adaptive Poisson–Boltzmann Solver) [61], at the modelled interface between hDHFR and hTYMS, showing a perfect complementarity. Partially positive or negative regions are indicated in blue and red, respectively. (C) Scheme of the final constructs differing only for the linker length and consequently named Chimera‐Long and Chimera‐Short. Structural renderings were produced using pymol.

To understand how DHFR and TYMS interact with each other and to design a chimeric fusion polypeptide including both proteins (hereafter called: Chimera), bifunctional enzymes endogenously expressed in several protozoa have been taken as an example. In particular, two classes of DHFR‐TYMS bifunctional enzymes can be distinguished, which differ mainly in the length of the linker between the two domains and consequently in the orientation with which the DHFR domain contacts the TYMS domain [33]. The crystallographic structure of DHFR‐TYMS from *Trypanosoma cruzi* [Protein Data Bank (PDB) ID: 2H2Q) [34] was chosen as a representative for the short linker class, whereas the structure from *Babesia bovis* (PDB ID: 3I3R) [35] was selected among several DHFR‐TYMS structures with long linkers from different organisms, as a result of the higher identity with the human DHFR sequence (33% and 55% similarity, respectively). The crystallographic structures of human DHFR and TYMS were aligned to the two representatives of the bifunctional enzymes to identify the two possible interaction interfaces. An analysis of the evolutionarily conserved residues at the interfaces showed that, in both cases, none of the residues involved in the stabilisation of the DHFR‐TYMS complex was conserved. Following this evidence, the surface potential at the interface was evaluated and a perfect complementarity of the surface charge of human proteins was revealed at the level of the interaction interfaces originating after a structural alignment with *B. bovis* DHFR‐TYMS (Fig. 5A,B). This suggests that the contacts between DHFR and TYMS are nonspecific and that the interaction interface on TYMS provides only an attractive force for DHFR and not an orienting one, as suggested previously [33].

Once the interaction modality was chosen, the following step was to design the linker. Even though, in the selected interaction mode, the C‐terminus of DHFR and the N‐terminus of TYMS are spatially near to each other, a knot would be created by joining them directly, which could prevent the Chimera fusion protein from folding correctly. For this reason, two different linkers were designed, a short 10 amino acids linker and a long 20 amino acids linker. The linkers were designed to be flexible but with a higher content of Ser residues compared to the canonical (GGGGS)_n_ flexible module [36] because bioinformatic analysis showed that the linker would be completely exposed to the solvent. Moreover, a sequence sensitive to digestion by PreScission protease was introduced to allow the separation of the two enzymes *in vitro* when needed. The two final linkers were GSSGGGSLFQGPSGGGSSGG for Chimera‐Long and GSLFQGPSGG for Chimera‐Short (Fig. 5C). The 3D structure of the designed bifunctional enzymes was then modelled based on homology using as templates the structures of hDHFR and hTYMS separated but aligned with the bifunctional enzyme from *B. bovis*. Finally, we used the crystallographic structure of TYMS from *Mus musculus* to model the N‐terminal residues of hTYMS, which is flexible and does not appear in any of the crystallographic structures from *Homo sapiens* available in the PDB.

### Expression, purification, biochemical and biophysical characterization of the chimeric constructs

**Fig. 6:**
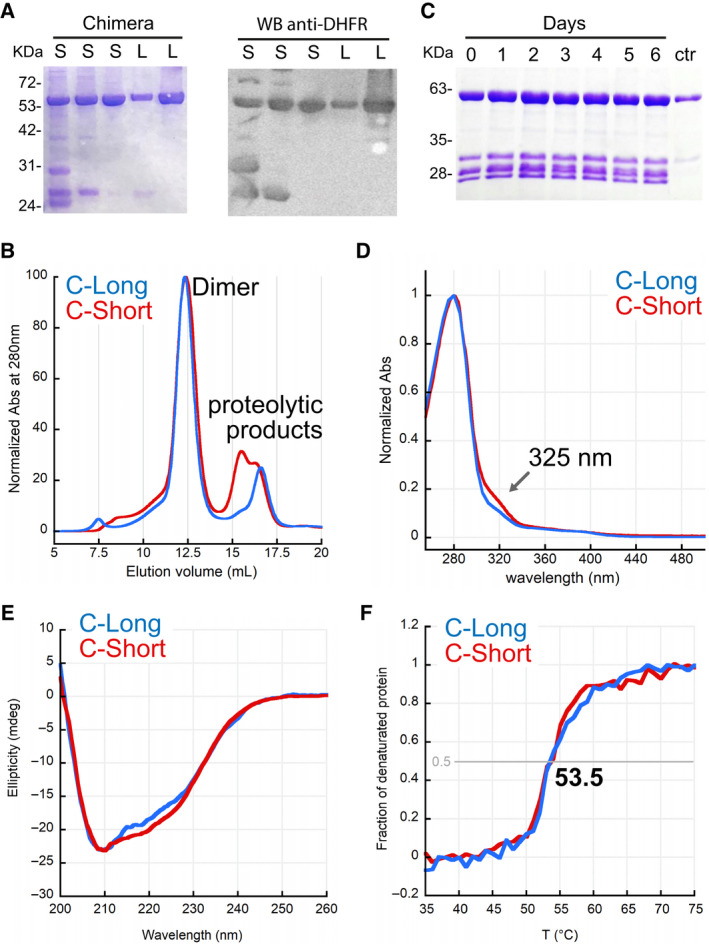
Purification and spectroscopic characterization of Chimera. (A) SDS/PAGE and western blotting of IMAC elution peaks (150–200 mm imidazole) of different preparations of Chimera‐Short (S) and ‐Long (L). (B) Typical SEC chromatogram of IMAC fractions of Chimera Constructs. The proteolyzed proteins are well separated from the main peak, containing the full‐length protein eluting as a dimer. The signal is normalized for a better comparison (column: Superdex 200 10/300). (C) Auto proteolysis assay: after IMAC, a small amount of Chimera‐Short was kept at 4 °C for 6 days to test whether the purified protein undergoes proteolysis in the purification buffer. Ctr, protein after SEC. No further proteolysis was observed. Typical final yields were between 6 and 4 mg·L^−1^ of culture for Chimera‐Long and ‐Short, respectively. (D) Normalised UV spectra of Chimera‐constructs in 100 mm potassium phosphate, pH 7.4. The shoulder at 325 nm is likely a result of bound NADPH. (E) Dichroic spectra of 10 µm Chimera constructs at 20 °C in a 1‐mm quartz cuvette. (F) Normalized thermal denaturation profiles of 10 µm Chimera constructs.

Both constructs were expressed with an N‐terminal histidine‐tag and were purified by immobilized metal affinity chromatography (IMAC), eluting between 150 mm and 200 mm imidazole. The constructs showed to undergo proteolysis in the linker region, as highlighted by the low molecular weight species detected by SDS/PAGE and western blotting of the purified fractions (Fig. 6A). This phenomenon was more evident at pH higher than 7.5 and was more pronounced for Chimera‐Short. To remove the proteolyzed protein, the fractions containing the two constructs were concentrated and further purified by size exclusion chromatography (SEC). Both Chimera‐Long and ‐Short eluted as dimers (Fig. 6B), suggesting that at least TYMS was correctly folded in the Chimera because human TYMS is dimeric. After purification, no further proteolysis occurred, and the proteins were stable for at least 1 week at 4 °C (Fig. 6C). The UV spectra of Chimera showed a shoulder around 325 nm, likely a result of NADPH bound to DHFR (Fig. 6D). This result suggested that also DHFR was correctly folded in the Chimera. Analysis by CD spectroscopy confirmed that both Chimera‐Short and ‐Long are folded and stable up to 45 °C, with an apparent melting temperature (*T*_m_) of 53.5 °C (Fig. 6E,F).

### Activity of Chimera and replication of the full thymidylate cycle *in vitro*

**Fig. 7:**
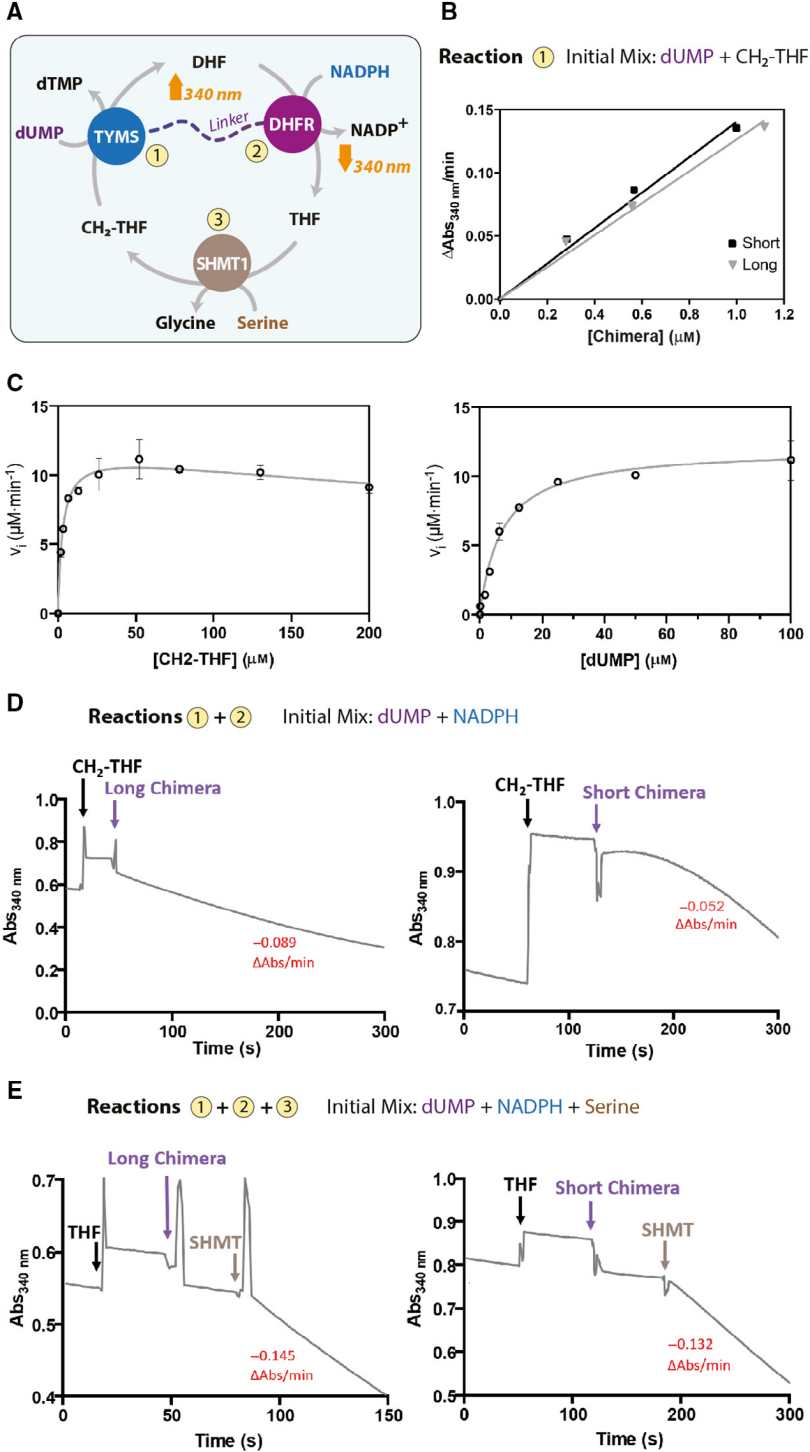
Activity assays: restoring the thymidylate cycle *in vitro*. (A) Scheme of the reactions assayed to test the catalytic activity of Chimera. All the reactions were performed at 20 °C in 20 mm K‐phosphate pH 7.2, 75 mm β‐mercaptoethanol. (B) Reaction 1; plot of the initial rate of dTMP formation as a function of Chimera concentration at constant substrate (0.1 mm dUMP, 0.2 mm CH_2_‐THF) (Chimera‐Short, black squares; Chimera‐Long, gray triangles). (C) Plot of initial rates of reaction 1 as a function of CH_2_‐THF concentration (left; 0.1 mm dUMP fixed concentration) and of dUMP concentration (right; 0.05 mm CH_2_‐THF fixed concentration). Error bars represent the SD of three independent measurements. (D) Reactions 1 + 2; time course of the coupled reactions of TMYS and DHRF. The observed rates (ΔAbs@340 nm·min^−1^) are also reported in red. To 0.1 mm dUMP and 0.1 mm NADPH, 0.1 mm CH_2_‐THF and 0.5 μm Chimera‐Long (left) or Chimera‐Short (right) were sequentially added (arrows). (E) Reactions 1 + 2 + 3; to the reaction mixture containing 0.1 mm dUMP, 10 mm serine and 0.1 mm NADPH, 16 μm THF and 0.5 μm Chimera‐Long (left) or Chimera‐Short (right) were added, and finally 0.5 μm SHMT1 was added (arrows). The thymidylate cycle can only start after the addition of SHMT1 that is needed to convert THF to CH_2_‐THF.

As a final quality control, the complete thymidylate cycle was analysed to assess the full functionality of the fused enzymes, as shown in Fig. 7. In a first assay, the reductive methylation of dUMP to dTMP catalysed by TYMS was assessed by following the increasing absorbance at 340 nm as a result of the formation of DHF from CH_2_‐THF (reaction 1; Fig. 7A). By increasing the enzyme concentration at constant substrates concentrations, a linear increase of TYMS activity is observed for both Chimera‐Short and ‐Long (Fig. 7B). However, because DHFR is fused to the N‐terminus of TYMS, and mutation in this region are known to affect catalysis [37, 38], as a control, we also performed a complete characterization of TYMS activity that yielded kinetic parameters (*K*_cat_ = 0.5 s^‐1^; *K*_m_ for CH_2_‐THF = 2.9 ± 0.5 μm; *K*_m_ for dUMP = 7.1 ± 1.0 μm) very similar to those reported for wild‐type TYMS [39] (Fig. 7C), indicating that TYMS is correctly folded and fully functional in the designed constructs.

Then, the DHFR activity of Chimera was investigated in a coupled assay, in which the DHF produced by TYMS activity is reduced to THF by DHFR, with concomitant NADPH oxidation (reactions 1 and 2; Fig. 7A). The reaction is observed by following the decrease of absorbance at 340 nm as a result of NADPH oxidation. The time course of the reaction steps is shown in Fig. 7D. With this experimental set‐up, both constructs were able to catalyse the coupled reactions at similar rates, indicating that DHFR is correctly folded and functional.

Finally, the complete thymidylate cycle was tested. The initial assay mix contained dUMP, serine and NADPH; then THF, Chimera and finally SHMT1 were added. In this experiment, as shown in Fig. 7E, the Chimera activity can start only when the substrate CH_2_‐THF is produced by SHMT1, the reaction proceeds until dUMP and NADPH are consumed, whereas the folate species cycles, thus reconstituting the functionality of the thymidylate cycle *in vitro*.

### *In* *vitro* analysis of the dTMP synthesis complex

The formation of dTMP‐SC between Chimera and SHMT1 was initially investigated by far‐western blotting (FWB) and IP. The dissociation constant was estimated by surface plasmon resonance (SPR) and bio‐layer interferometry (BLI) and, finally, the effect of substrates on complex formation was examined by differential scanning fluorimetry (DSF).

#### Detection of the complex by FWB and immunopurification assay

**Fig. 8:**
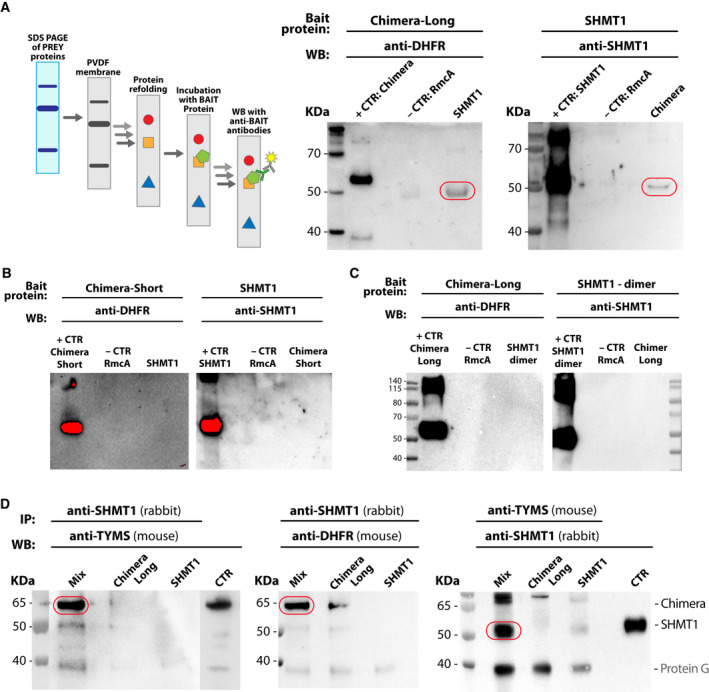
IP and FWB. (A) FWB: The prey proteins (SHMT1 or Chimera‐Long) were resolved alone by SDS/PAGE, electroblotted on a PVDF membrane, refolded and then incubated with 50 ng·mL^−1^ of the bait protein at 4 °C o.n. Left: the band detected at SHMT1 lane and height represents Chimera, and the band detected at Chimera lane and height, on the right image represents SHM1. The bait proteins were used in place of the prey proteins as positive controls, whereas RmcA (a bi‐domain bacterial protein construct from *P. aeruginosa* for which the two domains have a molecular weight comparable to DHFR and TYMS) was used as a negative control [63]. The formation of the complex between SHMT1 and Chimera‐Short (B) or SHMT1 dimeric mutant and Chimera‐Long (C) was tested by following the experimental set‐up described previously. Nevertheless, and despite over exposition of the membranes, in both cases, formation of the complex was not observed. (D) IP experiment: SHMT1 and Chimera‐Long were mixed at a ratio of 1 : 2 at a final concentration of 18 μm for SHMT1 and 36 μm for Chimera‐Long. The proteins were attached to the Protein G‐agarose beads by using specific antibodies (anti‐TYMS or anti‐SHMT1). The samples were incubated at 4 °C o.n. In the first two images (left), the detected band represents Chimera‐Long, whereas, in the image on the right, the detected band refers to SHMT1.

Formation of the complex between SHMT1 and the Chimera construct was initially evaluated by FWB assay. In this experiment, the purified Chimera constructs and SHMT1 were resolved by SDS/PAGE and electroblotted on a poly(vinylidene difluoride) (PVDF) membrane. The proteins were then renatured directly on the membrane and incubated with the bait protein (either SHMT1 or Chimera). In this way, Chimera and SHMT1 can form a complex in native conditions on the membrane that can be detected by specific antibodies (Fig. 8A). As shown in Fig. 8A, the antibodies directed against DHFR and SHMT1 detected the presence of Chimera‐Long in the lane of SHMT1 and vice versa. These results indicate that Chimera‐Long and SHMT1 interact with each other, but not with the control protein. The same results were not observed when using the Chimera‐Short construct (Fig. 8B), suggesting that the shorter linker might prevent the optimal orientation of TYMS and DHFR with respect to SHMT1. To further investigate the stoichiometry of the dTMP‐SC complex, the FWB was repeated using Chimera‐Long and a dimeric variant of SHMT1 (H135N‐R137A) [28]. The inability of dimeric SHMT1 to form the complex under the same experimental conditions (Fig 8C) clearly indicates that the tetrameric state of SHMT1 is crucial for complex formation.

The assembly of the dTMP‐SC was then confirmed by IP, which showed that SHMT1 and Chimera‐Long are interacting *in vitro* (Fig. 8D). The proteins were bound to the protein G‐agarose beads using either anti‐SHMT1 or anti‐TYMS antibodies; Chimera and SHMT1 were only present in the SHMT1 plus Chimera mixture (MIX sample), when detecting, respectively, with anti‐DHFR or anti‐TYMS and with anti‐SHMT1 antibodies, but not in the individual SHMT1 or Chimera samples (Fig. 8D).

As a result of these observations and the higher degree of purity and lower tendency to proteolysis of the Chimera‐Long construct, we chose to proceed using only this construct for further analysis of complex formation. Here after, if not otherwise specified, we refer to the Long construct as Chimera.

#### Quantification of the dissociation constant of the complex

**Fig. 9:**
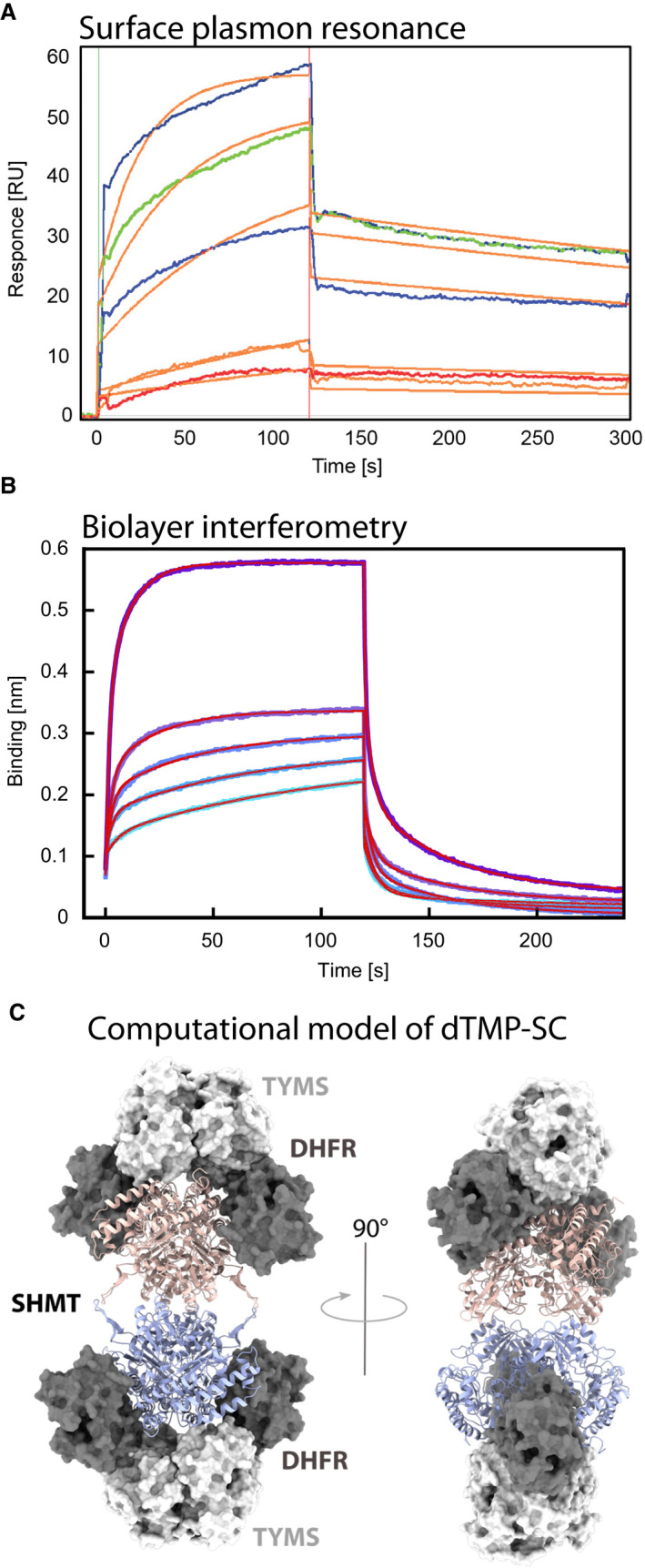
SPR and BLI experiments. (A) SPR: full kinetic analysis of SHMT1 (85, 42.5, 21.2, 5.3 and 2.66 μm) binding to Chimera (channel 3). Orange lines represent the global fits of the data to a 1 : 1 bimolecular interaction model. (B) BLI: aligned traces showing association and dissociation steps of SHMT1 (1, 3, 7.5, 15 and 30 µm) to Chimera, immobilized using mouse anti‐DHFR and a Protein‐A coated biosensor (in blue); time courses of each step were fitted with a two‐exponential equation (in red). The kinetic binding parameters [affinity (*K*_d_) and rate constants (*k*_on_, *k*_off_)] for the SHMT1‐Chimera interaction calculated from the SPR and BLI experiments are reported in Table 1. (C) Computational model of the dTMP‐SC: predicted interaction mode between human TS‐DHFR (light and dark grey surface) and SHMT1 tetramer (each dimer is depicted as pink and slate cartoons). Structural renderings were produced using pymol.

After evidence of complex formation, we performed SPR experiments to evaluate the binding affinity. We chose to immobilize Chimera and to use tetrameric SHMT1 as the analyte. Even though the precise stoichiometry of the putative complex is unknown, we previously showed that the dimeric SHMT1 variant is unable to form the complex. In addition, the higher symmetry of SHMT1 suggests that two Chimera dimers (DFHR‐TYMS:TYMS‐DHFR) may bind to one SHMT1 tetramer. In this case, the tetrameric form of SHMT1 would have two identical binding sites. This would allow the analyte to bind first with one site to the ligand Chimera and then to bind to another Chimera molecule in close contact to the second ligand site. The second binding event will give a stabilization of the ligand–analyte complex without extra response but shifts the equilibrium constant to a more stable interaction. This effect, which is often referred to as *avidity*, was observed in the case of SHMT1 binding to Chimera, suggesting that indeed SHMT1 may bind more than one Chimera dimer (Fig. 9A). A bivalent analyte gives rise to two sets of rate constants, one for each binding step, although the meaning of the two sets of rate constants and particularly the second set is very difficult to interpret. However, the avidity effects can be reduced using very low ligand levels and high analyte concentrations. Low ligand levels give a sparsely distributed ligand on the chip, with less chance of two ligand molecules being within reach of a single analyte. On the other hand, a high analyte concentration competes out the second binding site, favouring the formation of 1 : 1 complex. For this reason, we used a very low immobilization density of the ligand (175 resonance units on Channel 3) and high analyte concentrations (ranging from 85 to 2.7 μm). In this way, we minimized the avidity effect and used a 1 : 1 bimolecular interaction model to fit the experimental curves and estimate an initial value for the *K*_d_ of 2.5 and 2.9 μm on Channel 1 and 3 of the chip, respectively. This data show that the interaction between Chimera and SHMT1 is specific, and that the dissociation constant is in the low micromolar range. However, because we have previously shown that Chimera‐short is unable to bind SHMT1 as a result of the shorter linker, the possibility that the covalent immobilization on the SPR chip may also limit the conformational space of Chimera during binding must be taken into consideration. Therefore, BLI was used as alternative approach to gain more quantitative and reliable data. Chimera was immobilised using anti‐DHFR antibodies and a Protein‐A conjugated biosensor. SHMT1 was the analyte. We used the kinetic titration series experimental set‐up [40] to minimize background effects as a result of the multiple binding partners (Protein‐A, anti‐DHFR, Chimera) and kept the amount of immobilized ligand as constant as possible. Under these experimental conditions, both the association and the dissociation kinetics were biphasic, with the association rates dependent on SHMT1 concentration; data were fitted with a two‐exponential equation for each kinetics, assuming a general heterogeneous binding model in which two different binding events may occur (Fig. 9B). This interpretation gave a good fit, yielding the parameters reported in Table 1. The two binding events show comparable affinity (*K*_d_ ≈ 14 µm), suggesting that a symmetric bidentate model is likely the best interpretation for these data (Fig. 9B).

**Table 1:** Kinetic binding parameters calculated by SPR and BLI.

	SPR	BLI – binding 1	BLI – binding 2
*k*_on_ (M^−1^·s^−1^)	(4.7 ± 0.1) × 10^2^	(1.4 ± 0.1) × 10^4^	(1.6 ± 0.3) × 10^3^
*k*_off_ (s^−1^)	(2.5 ± 0.1) × 10^−3^	(2 ± 0.1) × 10^−1^	(2 ± 0.2) × 10^−2^
*k*_d_ (µm)	2.5 ± 0.4	16.4 ± 1.7	13.5 ± 3.6

Given that both SPR and BLI suggested that SHMT1 may bind two Chimera dimers, we performed a molecular docking starting from the available crystal structures of the three proteins that confirmed that this stoichiometry is actually feasible (Fig. 9C) and also that SHMT1 can indeed act as a bidentate analyte.

### Effect of substrates and ligands on dTMP‐SC formation

**Fig. 10:**
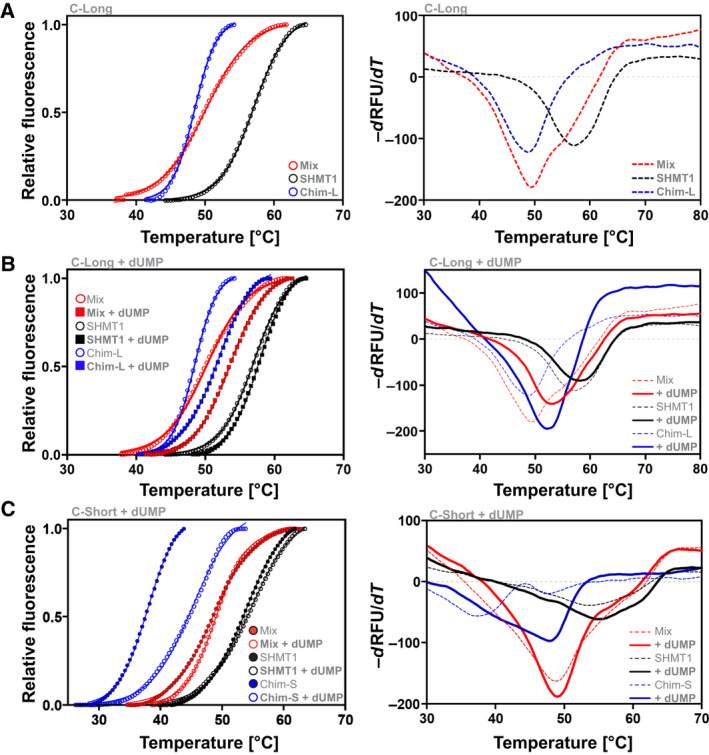
DSF. SHMT1 and Chimera constructs were mixed at a final concentration of 0.5 μm. When present, the concentration of CHO‐THF and NADPH were 0.1 mm, dUMP was 1 mm. All samples were incubated o.n. at 4 °C in 20 mm Na Hepes, pH 7.5, and 50 mm NaCl, before starting the analysis. (A) denaturation profiles of SHMT1, Chimera‐Long and of the mixed proteins in the absence of ligands or substrates. (B) Denaturation profiles of SHMT1 and Chimera‐Long in the presence of dUMP (square markers and continuous lines) compared to the signals obtained with no ligands/substrates (circle markers and dashed lines). (C) The same profiles as in (B) with the Short instead of Long construct. For all panels, plots in the left column show the change in fluorescence as a function of temperature, whereas, in the right column, the denaturation profiles are plotted as the first derivative of the fluorescence emission as a function of temperature. The calculated *T*_m_ for all the substrates assayed are reported in Table 2.

The effect of substrate/ligands on *in vitro* complex formation was assessed using DSF. Purified SHMT1 and Chimera were mixed and samples incubated overnight (o.n.). SHMT1 alone shows a *T*_m_ of 57.1 °C ± 0.1 °C, whereas the *T*_m_ of Chimera is 48.5 °C ± 0.5 °C (Table 2). When the two proteins were mixed, the observed *T*_m_ was 50.2 °C ± 0.1 °C (Fig. 10A). The change in the denaturation profile is likely a result of the interaction taking place between SHMT1 and Chimera. The effect of the presence of substrates, such as 5‐formyl‐tetrahydrofolate (CHO‐THF), dUMP, and NADPH was also tested. Although CHO‐THF and NADPH had no significant effect, dUMP stabilized Chimera and therefore the Chimera–SHMT1 complex, leaving the *T*_m_ of SHMT1 unaffected (Fig. 10B and Table 2). Moreover, the slope of the transition increases for the complex indicating that the denaturation process is more cooperative in the presence of dUMP. To confirm that the change in the denaturation profile observed in the presence of dUMP is a result of complex formation we performed the same experiment using the Chimera‐Short construct that fails to form the complex. In this case, the presence of dUMP, despite stabilizing Chimera‐Short (which as expected displayed an overall lower stability), has no significant effect on the stability of the mixed sample (Fig. 10C).

**Table 2:** Substrate effect on complex thermal stability: *T*_m_ (°C).

	SHMT1	Chimera‐Long	Mix
Buffer	57.1 ± 0.1	48.5 ± 0.5	50.2 ± 0.1
CHO‐THF	57.7 ± 0.2	48.2 ± 0.5	50.5 ± 0.2
NADPH	57.3 ± 0.5	48.3 ± 0.1	50.7 ± 0.1
dUMP	57.8 ± 0.7	51.8 ± 0.3	53.6 ± 0.1

	SHMT1	Chimera‐Short	Mix
Buffer	53.8 ± 0.7	37.9 ± 0.2	48.5 ± 0.2
CHO‐THF	54.0 ± 1.0	40.6 ± 0.3	48.0 ± 0.2
NADPH	53.6 ± 0.4	39.5 ± 0.2	47.0 ± 0.5
dUMP	54.7 ± 0.2	45.2 ± 0.2	48.9 ± 0.3

## Discussion

Despite its functional importance, nucleotide metabolism has received much less attention than genomics and protein expression studies, although the multiple metabolic enzymes involved in these pathways are currently chemotherapy targets [41]. The synthesis of most dNTPs occurs in the cytoplasm using the enzyme ribonucleotide reductase, whereas the formation of the DNA‐unique base dTMP relies on the concerted action of the TYMS, DHFR and SHMT enzymes forming the dTMP synthesis complex (dTMP‐SC). The dTMP‐SC was detected in the nucleus, anchored to the lamina, as well as in the mitochondria [6, 7]. Defects in dTMP synthesis increase genomic uracil because of replicative incorporation of dUMP instead of dTMP [12, 28, 30]. In addition, the dTMP synthesis pathway is essential for the replication of viral DNA, as shown for human herpesviruses, which may encode their own TYMS counterpart or upregulate the expression of cellular TYMS and DHFR [42, 43].

Here, we provide evidence that the three proteins involved in *de novo* dTMP synthesis assemble to form the dTMP‐SC in the cytoplasm and not only after nuclear translocation, as was assumed. The choice to use is‐PLA, with respect to co‐IP, allowed us to obtain novel information on space and time localization of the complexes. The signal of the dTMP‐SC complex was expected mainly in the nucleus, but only a slight increase of the nuclear complex was observed when S‐phase synchronised A549 cells were assayed. So far, we did not explore other conditions that were reported to increment nuclear complex formation, such as DNA damage, because this was beyond the scope of the present study.

**Scheme 2:**
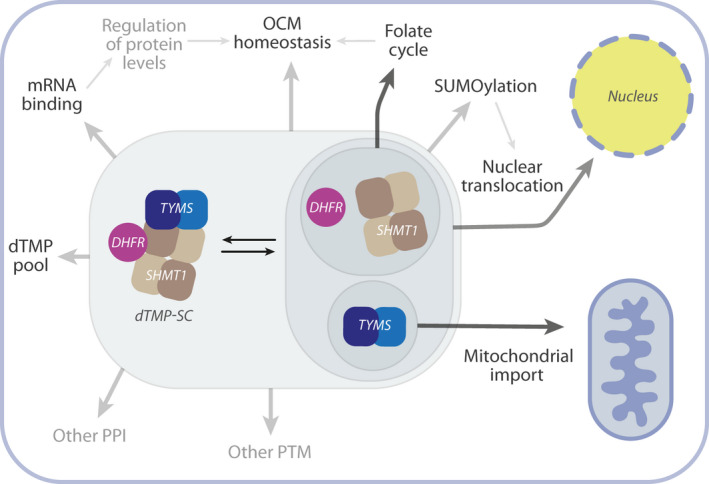
dTMP‐SC formation equilibrium in the cytosol. Scheme of the processes that may be affected by the assembly of the dTMP‐SC in the cytosol. After complex formation, some regions of the three enzymes may become less accessible. This may in turn affect: (A) the SUMOylation/deSUMOylation equilibrium, which controls nuclear translocation and possibly other functions; (B) the ability of the proteins to bind mRNA and cross‐regulate protein homeostasis and, in the case of SHMT1, also the catalytic activity (riboregulation) [45] with a direct effect on the folate cycle and OCM; (C) other PPI or post‐transcriptional modifications, as well as the mitochondrial import of TYMS.

The finding that dTMP‐SC can assemble in the cytoplasm has many implications, as summarised in Scheme 2, which will require further investigation. First, in the cytoplasm, SHMT1 and DHFR also participate in the folate cycle, whereas TYMS was shown to take part in mitochondrial *de novo* dTMP synthesis [6]; therefore, it is likely that formation of the dTMP‐SC complex may affect not only the dTMP pool, but also the whole one‐carbon metabolism. In addition, given that the complex certainly has a very different surface accessibility with respect to the single enzymes, it is probable that its assembly directly affects/controls other post‐transcriptional modifications, such as SUMOylation or the ability of these enzymes to bind RNA [27, 44, 45, 46].

dTMP‐SC formation may indeed represent a strategy to compartmentalize the enzymes in the cytosol and the effects of stabilization/destabilization of the complex should be investigated because this may represent an innovative and powerful tool for undermining the rewiring of the one‐carbon metabolism that tumour cells use to sustain proliferation. The evidence that the dTMP‐SC is abundant in the cytoplasm also suggests that the presence of DNA is not necessary to trigger complex assembly, allowing us to start an *in vitro* characterization of the complex, which is a prerequisite for future structural characterization and rational drug design.

Complex formation was indeed observed by FWB and IP analysis (Fig. 8). Interestingly, because the Chimera‐short construct failed to form the complex, it may be speculated that a longer flexible linker is necessary for TYMS and DHFR to reorient with respect to SHMT1. A negative effect of the short linker was also observed in the DSF experiment, where Chimera‐short failed to display the same stabilization effect as Chimera‐long in the presence of dUMP (Fig. 10). The dissociation constant (*K*_d_) of the Chimera and SHMT1 complex was estimated to be in the low micromolar range (Fig. 9), indicating that the dTMP‐SC complex is likely a transient assembly. We also observed a very clear avidity effect, which likely depends on the ability of SHMT1 to act as a bidentate binder. This is not surprising given that the tetrameric assembly of SHMT1 results in a higher symmetry with respect to Chimera that is dimeric. It is therefore possible that one SHMT1 tetramer binds to two Chimera dimers, as also confirmed by modelling the interaction. The tetrameric assembly appears to be crucial in the binding process because a dimeric variant of SHMT1 did not form the complex. Interestingly, although the minimal catalytic unit of SHMT1 is the dimer [28, 47] SHMT1 is tetrameric only in higher organisms [48]. Together with the increased affinity displayed for polyglutamylated‐THF [49, 50], the results of the present study suggest that the higher oligomeric state of SHMT1 may have evolved to favour novel protein–protein interactions, thus increasing the complexity of the cellular regulation network. The importance of the aggregation state in the control of the interaction between the SHMT isoforms and other binding partners was previously highlighted by us and other groups [28, 51, 52, 53]. The unique propensity of the SHMT2 isoform to form protein–protein complexes in the dimeric state [51], together with the evidence that SHMT1 forms the dTMP‐SC only when in the tetrameric form, as reported in the present study, further highlights that the two isoforms are indeed evolutionary distinct and fine‐tuned to achieve different goals in the cell, as well as strikingly optimized to minimize the superposition of tasks and interference between their specific cellular roles.

In the present study, the dTMP‐SC could be successfully assembled *in vitro* as a result of the Chimeric construct that we designed. Chimera was shown to be fully competent with respect to completing the thymidylate synthesis cycle when mixed with SHMT1 in the presence of NADPH, l‐serine and dUMP, after the addition of a catalytic amount of THF, with the folate species cycling until the total consumption of reducing equivalents (Fig. 7). This result provides a good starting point for further investigating the kinetic properties of the dTMP‐SC and eventually searching for possible inhibitors of the entire complex, which may significantly differ from those found for the individual components.

In conclusion, the characterization of the cellular dynamics and structural/functional properties of the dTMP‐SC reported in the present study provides a significant advance in our understanding of the role of this complex in normal and cancer cells. At this stage, it cannot be excluded that other proteins or binding partners may interact with the dTMP‐SC and modulate its assembly, including components of the replicase machinery in the nucleus [10] or other nucleic acids such as RNA in the cytosol. Intriguingly, both TYMS and DHFR bind to their own mRNA in the absence of substrates [46] and we recently demonstrated that SHMT1 also binds RNA, which inhibits enzymatic activity in a selective way [12, 27, 45]. Regulation of the metabolic activity by RNA molecules was recently also reported for enolase 1 [54], strongly suggesting that riboregulation of cellular metabolism may take unexpected routes. Undoubtedly, we are witnessing an era in which an increasing number of entangled regulatory mechanisms are emerging as being central for the cellular control system: weak PPI, riboregulation, miRNA, lncRNA and microproteins are just some examples of the actors shaping a regulatory network that is far more sophisticated than could be predicted. We belive that the dTMP‐SC assembly is certainly playing a crucial role in this network, especially in cancer cells. Identifying the missing component(s) will allow further characterization of the complex and advances in the structural analysis, which is certainly the most challenging goal.

## Materials and methods

### Chemicals and reagents

Thymidine (T1895), paraformaldehyde, sucrose, phosphate buffered saline (PBS), Tween 20, BSA, 4′,6‐diamidino‐2‐phenylindole (DAPI), mounting media, phenylmethanesulfonyl fluoride, deoxyribonuclease I from bovine pancreas, Hepes, MgCl_2_, NaCl, glycerol, Triton X‐100, imidazole, 2‐mercaptoethanol, dUMP, NADPH, l‐serine and Duolink PLA kit (DUO92007) were purchased from Sigma‐Aldrich. THF was provided from Merck & Cie (Schaffhausen, Switzerland). Protein G‐Plus agarose beads (Sc‐2002) were purchased from Santa Cruz Biotechnology, Inc. (Santa Cruz, CA, USA). Dulbecco’s phosphate buffered saline (20‐031‐CV), fetal bovine serum (35‐015‐CV) and 0.25% trypsin (25‐053‐CI) were purchased from Corning Inc. (Corning, NY, USA); BCA kit (quantumMicro Protein, EMP015480) was obtained from EuroClone (Pero, Italy).

### Antibodies

The antibodies used were: rabbit anti‐SHMT1 (HPA0233314; Atlas Antibodies, Stokholm, Sweden); mouse anti‐DHFR (WH0001719M1; Sigma‐Aldrich); rabbit anti‐TYMS (MBS126074; Mybiosource, San Diego, CA, USA) indicated as TYMS_R_; mouse anti‐TYMS (sc‐33679; Santa Cruz Biotechnology, Inc.) indicated as TYMS_M_; mouse anti‐PCNA Alexa Fluor® 488‐conjugated antibody (ab201672, PC10 clone; Abcam, Waltham, MA, USA), mouse anti‐α‐Tubulin (T5168, clone B512; Sigma‐Aldrich); mouse anti‐tubulin‐FITC (f2168; Sigma‐Aldrich); rabbit anti‐Histone‐H3 (H0164; Sigma‐Aldrich); mouse anti‐SHMT1 (sc‐365203; Santa Cruz Biotechnology, Inc.); rabbit anti‐SHMT1 (D3B3J, cell signaling technology); anti‐DHFR (sc‐377091; Santa Cruz Biotechnology, Inc.); anti‐chicken tubulin (ab89984; Abcam); anti‐mouse FITC (AB_2338; Jackson ImmunoResearch, West Grove, PA, USA); anti‐rabbit FITC (AB_2337977; Jackson ImmunoResearch); anti‐mouse Rhodamine (AB_23387;66 Jackson ImmunoResearch); anti‐rabbit CY3 (AB_2338000; Jackson ImmunoResearch); mouse anti‐rabbit IgG‐HRP (sc‐2357; Santa Cruz Biotechnology, Inc.); m‐IgGκ BP‐HRP (sc‐516102; Santa Cruz Biotechnology, Inc.); anti‐chicken Alexa Fluor 647 (703605155; Jackson ImmunoResearch); Duolink *is*‐pla probe anti‐mouse minus (duo92004‐100rxn; Sigma‐Aldrich); and Duolink *is*‐pla probe anti‐rabbit plus (duo92002‐100rxn; Sigma‐Aldrich).

### Cell lines

A549 lung cancer cell lines were purchased from ATCC (Manassas, VA, USA). The cells were grown in RPMI‐1640 medium (Corning Inc.), supplemented with 100 IU·mL^−1^ penicillin/streptomycin (P 4458; Sigma‐Aldrich) and 10% foetal bovine serum (Corning Inc.). HeLa cells (CCL‐2) were purchased from ATCC (Manassas, VA, USA), cultured in Dulbecco’s modified Eagle’s medium and supplemented with 2% penicillin/streptomycin, 2% l‐glutamine, 2.5% Hepes and 10% foetal bovine serum. All experiments were run in triplicate and in separate biological sets.

### Cell synchronization

Cells were synchronised in S‐phase by a single thymidine block. Cells were treated as follows: (a) 2 mm thymidine (T1895; Sigma‐Aldrich) for 24 h at 37 °C, to block DNA synthesis; (b) release in a thymidine‐free medium; (c) after 4 h, cells were fixed in 3.7% paraformaldehyde/30 mm sucrose for 10 min and processed either for the is‐PLA experiment using the Duolink PLA kit (DUO92007; Sigma‐Aldrich) in accordance with the manufacturer’s instructions, or for the IF.

### IF

IF staining was performed to check whether the cells were synchronised in S‐phase (Fig. 1D), and to assess the differential cellular localization of the single proteins (SHMT1, TYMS and DHFR) according to the cellular phase, as well as to co‐localize the proteins.

Asynchronous and S‐phase synchronised A549 (lung cancer cell lines) were grown on coverslips and fixed in 3.7% paraformaldehyde/30 mm sucrose for 10 min. For the co‐localization samples, asynchronous A549 were fixed using ice‐cold methanol and incubating the coverslips at −20 °C for 6 min. Afterwards, cells were permeabilized in 0.1% Triton X‐100 and blocked in 3% bovine serum albumin in PBS/0.05% Tween‐20 for 1 h at room temperature. The incubation with primary antibodies was performed at 4 °C o.n. in a dark humidity chamber. Subsequently, the fluorescence labelled secondary antibodies were added in PBS containing 0.05% Tween 20 and 3% BSA solution and incubated at room temperature for 30 min. Cells were counterstained with DAPI (0.1 μg·mL^−1^) and mounted using the mounting media.

Primary antibodies were: (a) rabbit anti‐SHMT1 (dilution 1 : 50); (b) mouse anti‐DHFR (dilution 1 : 50); (c) rabbit anti‐TYMS (dilution 1 : 20); (iv) anti‐PCNA Alexa Fluor® 488‐conjugated antibody (dilution 1 : 1000); (e) anti‐tubulin‐FITC (dilution 1 : 300); (f) anti‐chicken tubulin (dilution 1 : 50); and (g) mouse anti‐TYMS (dilution 1 : 50).

Secondary antibodies were: (a) anti‐mouse FITC (dilution 1 : 100); (b) anti‐rabbit FITC (dilution 1 : 50); (c) anti‐mouse rhodamine (dilution 1 : 50); (d) anti‐rabbit CY3 (dilution 1 : 500); and (e) anti‐chicken Alexa Fluor 647 (dilution 1 : 100).

### is‐PLA

*is*‐PLA was performed in both synchronised and asynchronous A549 and HeLa cell lines using the Duolink PLA kit and in accordance with the manufacturer’s instruction. The primary antibodies pair to detect the interaction among the three proteins were: (a)mouse anti‐DHFR (dilution 1 : 50)/rabbit anti‐SHMT1 (dilution 1 : 50); (b) rabbit anti‐TYMS (dilution 1 : 20)/mouse anti‐DHFR (dilution 1 : 50); and (c) rabbit anti‐SHMT1 (dilution 1 : 50)/mouse anti‐TYMS (dilution 1 : 50)

The above‐mentioned antibodies were incubated o.n. in a dark humidity chamber at 4 °C. Subsequently, the incubation with the PLA probes was performed in a pre‐heated humidity chamber for 1 h at 37 °C followed by the ligation addition. If the target proteins (SHMT1‐DHFR‐TYMS) interact among each other or are very close, this step will produce a DNA circle. The amplification time was 100 min and was performed at 37 °C in a dark humidity chamber. Regarding negative controls: the PLA experiment was performed without one of the primary antibodies (Fig. 4B). DNA was stained with DAPI as described above.

#### siRNA cell transfection

Cells were seeded at the density of 10^5^ cells per well on six‐well culture plate in RPMI. Twenty‐four hours after seeding, cells were transfected with 15 nmol·L^−1^ siRNA with AllStars RNAi (Qiagen, Hilden, Germany). Controls scrambled sequences, or siRNA sequences against shmt1 (Qiagen) using specific siRNA sequences as described in [55] using the JetPRIME reagent (PolyPlus, Illkirch‐Graffenstaden, France), in accordance with the manufacturer’s instructions. After 48 h of transfection, the cells were detached and used for PLA experiments.

## Cell fractionation

Together with IF, cell fractionation was performed to analyse the cellular localization of the single proteins (SHMT1, TYMS and DHFR) according to the cellular phase.

Nuclear and cytosolic fractionation of A549 cell lines (both asynchronous and S‐phase enriched) was performed by treating with trypsin and washing the cells twice in ice‐cold PBS. Cells were lysed in 200 μL of cytoplasmic lysis buffer (10 mm Tris, pH 7.8, 1.5 mm MgCl_2_, 10 mm KCl, 1 mm phenylmethanesulfonyl fluoride) and kept on ice for 10 min. Nuclei were then pelleted at 1000 ***g*** for 7 min at 4 °C, and the supernatant containing the cytoplasmic fraction was removed. Nuclei were resuspended in 60 μL of SDS 10% incubated for 30 min on ice, boiled for 10 min and the DNA fraction was sedimented by centrifugation at 1000 ***g*** for 5 min at 4 °C. Protein concentrations were determined by the BCA assay. Cytosolic and nuclear fractions (20 µg) were separated by SDS/PAGE and transferred into a nitrocellulose membrane. Western blot analysis was performed as usual, and the primary antibodies were diluted 1 : 1000. Western blotting was performed using mouse anti‐SHMT1 (dilution 1 : 1000; Cell Signaling, Danvers, MA, USA), mouse anti‐DHFR (dilution 1 : 1000) and rabbit anti‐TYMS (dilution 1 : 1000). Anti‐tubulin (cytosolic marker; dilution 1 : 1000) and anti‐Histone H3 (nuclear marker; dilution 1 : 5000) were used as controls.

## Microscopy analysis on fixed samples

Samples were acquired using an Eclipse 90i microscope (Nikon Instruments S.p.A., Campi Bisenzio Firenze, IT, USA) equipped with 40× (N.A. 0.75) and 100× (oil immersion, N.A. 1.3) objectives and a Qicam Fast 1394 CCD camera (QImaging Surrey, BC, Canada) or with an inverted microscope (Eclipse Ti; Nikon) using a 60× (oil immersion, N.A. 1.4) objective and the Clara camera (ANDOR Technology, Belfast, UK). Images were acquired using nis‐elements ar 3.2 (Nikon Instruments S.p.A.) or nis‐elements hc 5.11 using the JOBS module for automated acquisitions; elaboration and processing was performed using nis‐elements hc 5.02 (Nikon Instruments S.p.A.) and gimp ‐ gnu image manipulation program.

## Quantitative analyses of fluorescence signals

For immunofluorescence quantification of fluorescence intensity signals, images of interphase cells of asynchronous and S‐enriched populations were acquired using 60× or 40× objectives, along the z‐axis every 0.4 μm for 5 μm. Signals were measured using nis elements hc 5.02 (nd2 file format) (Nikon Instruments S.p.A.) as: sum intensity values of the nuclear fluorescence with respect to sum intensity values of the cytoplasmic fluorescence. To avoid differences as a result of different cell sizes, for each cell, the same square mask was used to calculate the nuclear and the cytoplasmic fluorescence. Images were corrected for external background. For is‐PLA spots of interaction counts, images were acquired with a 60× objective, along the z‐axis every 0.4 μm for 8 μm. The ‘general analysis’ module of nis elements hc 5.11 was then used for automatic spot count; is‐PLA signals were identified based on fixed parameters in all images and those inside the nuclei (defined by DAPI signal) were counted.

Images for quantification fluorescence signals were maximum intensity projections from *z*‐stacks (0.4 μm for a range of 5–8 μm). Statistical analyses were performed with prism 8 (GraphPad Software Inc., San Diego, CA, USA).

## Chimera rational design

Structural analysis necessary to design and model the two constructs of Chimera was largely conducted within pymol (The PyMOL Molecular Graphic System, version 1.8; Schrödinger, LLC, New York, NY, USA) taking advantage of the pymod 2.0 plugin [56] that allows the performance of sequence and structural alignments as well as homology modelling by integrating blast, clustal omega, muscle, cealign and modeller [57] into pymol. The structure of DHRF‐TYMS from *B. bovis* (PDB ID: 3I3R) was used as a reference to orient the structures of human DHFR and TYMS (PDB ID: 1DRF; 1HZW [58, 59]), the linker regions were modelled using the *de novo* loop modelling function in modeller, and the N‐terminal portion of hTYMS was modelled based on the closest homologous structure (TYMS from *M. musculus*; PDB ID: 4EB4 [60]). The final Chimera models were further refined using the sculpting function in pymol and moe [Molecular Operating Environment (MOE), 2019.01; Chemical Computing Group ULC, Montreal, QC, Canada] using the conjugate gradients method for energy minimization. Surface electrostatic potentials were calculated using apbs (Adaptive Poisson‐Boltzmann Solver) [61], also available as a pymol plugin. Sequence conservation analysis was performed using campo [62].

## Protein expression and purification

### Chimera

Long and Short Chimera genes were synthesized and optimized for expression in *Escherichia coli* by GeneArt (Life Technologies, Carlsbad, CA, USA), cloned into a pET28a expression vector (Invitrogen, Carlsbad, CA, USA), using *Nde*I and *Xho*I restriction sites. Small scale expression tests were made on two different *E. coli* expression strains varying: isopropyl thio‐β‐d‐galactoside (IPTG) concentration; temperature and expression time; and lysis protocol (Table 3). The overexpressed fusion proteins were largely present in the inclusion bodies. However, good results, in terms of solubility and yield of protein, were obtained by basal expression of the protein in BL21(DE3) cells, grown at 25° for 24 h, without IPTG, and cell lysis by sonication. This protocol was therefore applied for larger scale expressions of both Chimera‐Long and ‐Short as N‐terminal His‐tagged fused proteins. Cells were harvested by centrifugation at 4 °C, resuspended in the lysis buffer (0.1 m K‐phosphate, pH 7.5; 1 mm phenylmethanesulfonyl fluoride; 4 mg·mL^−1^ deoxyribonuclease I from bovine pancreas; 5 mm MgCl_2_; 5% glycerol) and lysed by ultrasonic disruption on ice. The supernatant, supplemented with 10 mm imidazole, was purified by IMAC on a Ni^2+^‐His‐Trap column (GE Healthcare, Chicago, IL, USA) in buffer 0.1 m K‐phosphate pH 7.5, 10 mm imidazole. The IMAC fractions containing the protein were concentrated by ultrafiltration (Amicon; Millipore, Burlington, MA, USA) and then injected onto a Superdex 200 10/300 FPLC column (GE Healthcare) equilibrated with buffer K‐phosphate 0.1 m, pH 7.5, and eluted with a flow rate of 0.9 mL·min^−1^. The column was calibrated using the protein standards; aprotinin, carbonic anhydrase, ovalbumin, conalbumin, aldolase and ferritin (Sigma‐Aldrich): *r* ^2^ was 0.994. The purity of the protein was assessed by SDS/PAGE. The molar extinction coefficient at 280 nm was determined experimentally by the BCA assay (Sigma‐Aldrich) in accordance with the manufacturer’s specifications and was 1.51 (1 mg·mL^−1^) or 91355 m ^−1^·cm^−1^. The purified proteins were aliquoted, frozen in liquid nitrogen and stored at −20 °C until use.

### SHMT1

*SHMT1* was expressed and purified as described previously [53].

**Table 3:** Heterologous expression conditions assayed for Chimera‐Short and ‐Long.

Expression strain	Growth temperature (°C)	Expression temperature (°C)	Experiment time (h)	IPTG (mm)	Lysis method
BL21(DE3)	37 – 25	37 – 25 – 18	5 – 16 – 24	0 – 0.1 – 0.5	Sonication lysozyme
BL21(DE3)pLysS	37	18	5 – 16	0.1 – 0.5	Sonication lysozyme

## CD and thermal melting spectroscopic analysis

The CD spectra were collected, for both Short and Long Chimera, at a 10 μm protein concentration in a 0.1‐cm quartz cuvette (Hellma, Jena, Germany) using a J‐710 spectropolarimeter (Jasco, Tokyo, Japan) equipped with a Peltier temperature control unit. The thermal stability of the two proteins was measured through the thermal melting assay method of the instrument, by increasing the temperature from 30 °C to 90 °C with a 1 °C·min^−1^ rise in temperature, monitoring the dichroic signal at 220 nm every 0.5 °C.

## Activity assays

Activity assays were carried out at 20 °C in 20 mm K‐phosphate, pH 7.2, containing 75 mm 2‐mercaptoethanol, using a Hewlett‐Packard 8453 diode‐array spectrophotometer (Agilent Technologies, Santa Clara, CA, USA) and a 1‐cm pathlength cuvette. Regarding the TYMS activity assay, substrate concentration was 0.2 mm CH_2_‐THF, 0.1 mm dUMP and varying concentration of Chimera (0.3, 0.6 and 1.1 µm). Saturation curves, from which TYMS kinetic parameters were calculated, were obtained using 1 µm Chimera keeping one substrate at a fixed concentration (0.1 mm dUMP or 0.05 mm CH_2_‐THF) and varying CH_2_‐THF from 0 to 0.2 mm or dUMP from 0 to 0.1 mm. To calculate kinetic parameters, the saturation curve shown in Fig. 10C (left) was fitted to an equation describing the substrate inhibition [24], whereas the saturation curve in Fig. 10C (right) panel was fitted to the Michaelis–Menten equation. Concerning TYMS‐DHFR activity, substrate concentration was 0.1 mm CH_2_‐THF, 0.1 mm dUMP and 0.1 mm NADPH, whereas Chimera was 0.5 µm. In the activity assay of the full tymidilate cycle the substrate concentration was 10 mm l‐serine, 16 μm THF, 0.1 mm dUMP, 0.1 mm NADPH. Chimera and SHMT1 concentration was 0.5 µm.

## FWB

The prey [Chimera (4 µg), SHMT1 (4 µg)] and the control protein, a truncated construct of RmcA form *Pseudomonas aeruginosa* (4 µg) [63], SHMT1 or SHMT1‐dimeric mutant (H135N‐R137A; 0.5 µg), Chimera (0.5 µg)] were resolved by SDS/PAGE electrophoresis, and electro‐blotted onto PVDF membranes (Bio‐Rad, Hercules, CA, USA: 100 V, 2h, 4 °C). The transferred proteins were then renatured by progressively reducing the guanidine‐HCl concentration, starting from the following stock solution: AC buffer (10% glycerol; 5 m NaCl; 1 m Tris, pH 7.5; 0.5 m EDTA; 10% Tween‐20; 8 m guanidine‐HCl; 2% Milk; 1 mm dithiothreitol) as described by Wu *et al*. [64]. Proteins were renaturated by incubating the membranes for 30 min at room temperature in AC buffer containing decreasing concentrations of guanidine‐HCl (6 m and 3 m), then for 30 min at 4 °C in 0.1 m guanidine‐HCl and finally o.n. at 4 °C in AC buffer containing no guanidine‐HCl. The next day, the membranes were blocked with 5% milk in PBS‐Tween for 1 h at room temperature and incubated with purified bait proteins (SHMT1 or SHMT1‐dimeric mutant and Chimera 50 ng·mL^−1^) at 4 °C overnight. Membranes were then washed and incubated with primary antibody against the protein used for the incubation step [mouse anti‐DHFR (dilution 1 : 1000), mouse anti‐TYMS (dilution 1 : 1000) and rabbit anti‐SHMT1 (dilution 1 : 1000; Cell Signaling)] o.n. at 4 °C. Subsequently, membranes were incubated with the appropriate secondary antibody and detected with Luminata^?^ Crescendo Western HRP Substrate (Millipore) in accordance with the manufacturer’s instructions.

## Immunopurification with Protein G‐plus agarose beads

SHMT1 and Chimera‐Long were mixed at a ratio of 1 : 2 in 50 mm NaCl, 20 mm Hepes, pH 7.5. Each sample was 1 mL in volume and contained SHMT1 (40 μg), Chimera (92 μg) or the mixed proteins. A precleaning step of the samples was performed by adding 50 μL of Protein G‐Plus agarose beads suspension (Santa Cruz Biotechnology, Inc.) to 1 mL of each sample (Chimera, SHMT1 and Mix) and incubating for 2 h at 8 °C on a rocking platform. The beads were then centrifuged at 12 000 ***g*** for 20 s and the supernatant was transferred to fresh tubes. Afterwards, 1 μg of anti‐SHMT1 (Santa Cruz Biotechnology, Inc.) or anti‐TYMS (Santa Cruz Biotechnology, Inc.) was added to each sample, incubating for 1 h at 8 °C rocking. Next, 50 μL of beads were added to the mixture and the final solution was left rocking at 8 °C o.n.. Samples were centrifuged at 12 000 ***g*** for 20 s and the supernatant was discarded. Two washes were performed by resuspending the beads in 500 μL of 50 mm NaCl and 20 mm Hepes, pH 7.5, and rocking for 30 min at 8 °C. The supernatant was discarded. Then, 50 μL of SDS/PAGE loading buffer 2× were added to the beads‐protein mixture. Samples were then heated at 95 °C for 1 h, resolved by SDS/PAGE electrophoresis and subsequently electroblotted onto a nitrocellulose membrane following a standard western blot protocol. For the detection of the prey proteins mouse anti‐DHFR (dilution 1 : 1000), mouse anti‐TYMS (dilution 1 : 1000) and rabbit anti‐SHMT1 (dilution 1 : 1000; Cell Signaling) antibodies were used.

## SPR

SPR experiments were performed at 25 °C using a Pioneer AE optical biosensor (Molecular Devices‐ForteBio, Shanghai, China) equipped with a COOH2 sensor chip (short carboxylated polysaccharide coating) and equilibrated with running buffer 10 mm Hepes, pH 7.4, 150 mm NaCl, 0.005% Tween‐20. The COOH2 sensor chip was installed and conditioned in accordance with the manufacturer’s instructions. Then, the chip was chemically activated for 6 min by injecting 150 μL of a 1 : 1 mixture of 100 mm *N*‐hydroxysuccinimide and 400 mm ethyl‐3(3‐dimethylamino)propyl carbodiimide, diluted 1 : 10 with water, at a flow rate of 25 µL·min^−1^. Chimera‐Long was immobilized onto the activated sensor chip using standard amine‐coupling procedures [65]. Briefly, a 0.1 mg·mL^−1^ Chimera solution (in 10 mm sodium acetate, pH 4.5) was injected at a flow rate of 10 μL·min^−1^ on channels 1 and 3 (channel 2 was used as reference for non‐specific binding), followed by a 70‐μL injection of 1 m ethanolamine, pH 8.0, to block any remaining activated groups on the surface. Chimera was captured to a density of 735 and 175 resonance units on Ch1 and Ch3 channels, respectively. The stability of the Chimera surface was demonstrated by the flat baseline achieved at the beginning (0–60 s) of each sensorgram. The analyte SHMT1 was dialyzed in the running buffer and injected at different concentrations (85, 42.5, 21.2, 10.6, 5.3, 2.66 μm) onto the sensor chip at a constant flow rate of 75 µL·min^−1^. A dissociation of 180 s was allowed. At least two experiments were performed. The interaction of the analyte with immobilized Chimera was detected as a measure of the change in mass concentration, expressed in resonance units. All sensorgrams were processed using double referencing [66]. First, responses from the reference surface (Ch2) were subtracted from the binding responses collected over the reaction surfaces to correct for bulk refractive index changes between flow buffer and analyte sample. Second, the response from an average of the blank injections (zero analyte concentrations) was subtracted to compensate for drift and small differences between the active channel and the reference flow cell Ch2 [67]. To obtain kinetic rate constants and affinity constants, the corrected response data were fitted in qdat software provided with the instrument (Biologic Software, Canberra, Australia). A kinetic analysis of the ligand/analyte interaction was obtained by fitting the response data to a reversible 1 : 1 bimolecular interaction model. The equilibrium dissociation constant (*k*_d_) was determined by the ratio *k*_off_/*k*_on_. This experiment was performed in duplicate. Moreover, to exclude mass‐transfer, we replicated the same experiment at two different higher flow rates (75 µL·min^−1^ and 150 µL·min^−1^) obtaining substantially the same value of *K*_d_ (in the presence of mass transfer limitation we should have obtained different *K*_d_ values).

## BLI

Bio‐layer interferometry was performed using the BLItz platform (ForteBio). Protein‐A probes (ProA; 18‐5010; ForteBio) were used as biosensors, Chimera was immobilised using mouse anti‐DHFR (Sigma‐Aldrich), and SHMT1 was used as the analyte at different concentrations.

Several set‐ups were tried to minimize the background processes interfering with the kinetics; initially, experiments were carried out by changing the biosensor at each measurement. This more conventional approach was discarded as a result of the low reproducibility caused by both the avidity effect and the variability of the immobilized ligand in each step. We followed the ‘kinetic titration series’ protocol described by Frenzel *et al*. [68], which uses the same biosensor for all the titration steps. In brief: Protein‐A biosensor was hydrated in kinetic buffer (18‐1105; ForteBio) for 10 min. Afterwards, a mixture of anti‐DHFR (5 μg ml^−1^) and Chimera (4.5 µm) was loaded for 120 s at 2200 r.p.m shaking speed. The unbound antibody‐protein complex was washed away for 240 s with kinetic buffer. The anti‐DHFR‐Chimera loaded sensor was then incubated with SHMT1 at different concentrations (1, 3, 7.5, 15, 30 µm), with 120 s of association and 120 s of dissociation in kinetic buffer at 2200 r.p.m. This set‐up led to a biphasic behaviour on both the association and dissociation kinetics; because the vendor software includes automatic global fitting only for monophasic events, data were manually fitted with a two‐exponential equation with igor pro (Wavemetrics, Portland, OR, USA). For the dissociation time course, a very fast phase (including the first 10 s of kinetics) was occasionally observed at high SHMT1 concentrations. This is likely the result of a small percentage of loosely immobilised ligand that may dissociate from the biosensor after binding the analyte; if present, this part was not included in the fit. The two main dissociation phases were fitted separately and then the fit coefficients were imposed in the final two‐exponential fit. The observed *k*_off_ in the dissociation kinetics were used as constrain for *k*_on_ determination. The values reported in Table 1 were obtained by linear fit of the *k*_obs_, as obtained by two series of independent experiments, with each series including at least four different SHMT1 concentrations. A representative series is shown in Fig. 9B. Reference binding experiments were carried out titrating SHMT1 with a sensor loaded with the anti‐DHFR in the absence of Chimera. Operating temperature was maintained at 25 °C.

## Computational model of dTMP‐SC

Protein Structures preparation and docking: human TS (PDB ID: 1JU6 [69]), DHFR (PDB ID: 2DHF [70]) and SHMT1 (PDB ID: 1BJ4 [71]) were downloaded from the PDB. TS and DHFR were initially superposed to Chimera. moe [72] and pymod 3 [73] were used to add missing hydrogens and partial charges. The structures were energy minimized using default values in moe. To gain insight into their interaction mode, the DHFR‐TS complex was docked to SHMT1 using the haddock server (https://wenmr.science.uu.nl/haddock2.4) [74], a widely used protein–protein docking server. The server allows backbone conformational modification during complex formation. Default parameters were kept for docking. Ten different starting orientations of the complex were submitted, and the best one, as assessed by haddock score, was kept and is shown in Fig. 9C.

## DSF

DSF assays were performed using a real‐time PCR Instrument (CFX Connect Real Time PCR system; Bio‐Rad). In a typical experiment, 0.5 μm SHMT1 and 0.5 μm Chimera (Long or Short) in 20 mm Na Hepes, pH 7.2, containing 50 mm NaCl, were incubated o.n. at 4 °C (in a total volume of 25 μL) in a 96‐well PCR plate. When substrates were added, this solution also contained 0.1 mm CHO‐THF, 1 mm dUMP and 0.1 mm NADPH. After the incubation, Sypro Orange (4×; Thermo Scientific, Waltham, MA, USA) was added and fluorescence was measured from 25 °C to 95 °C in 0.4 °C/30 s steps (excitation 450–490 nm; detection 560–580 nm). All samples were run in triplicate. Denaturation profiles were analysed using prism, after removal of points representing quenching of the fluorescence signal as a result of post‐peak aggregation of protein–dye complexes. All curves were normalised and fitted to the following sigmoidal equation to obtain the melting temperatures (*T*_m_):

$$\text{Fluorescence}\;=\;F_{1}\;+\;\frac{\left(F_{2}\;‐\;F_{1}\right)}{1\;+\;e^{T_{m}‐\frac{X}{s}}}$$

where *X* is the temperature ( °C), *F*_1_ is the fluorescence at low temperature and *F*_2_ the maximal fluorescence at the top of the truncated dataset, whereas *s* describes the steepness of the curves. Alternatively, *T*_m_ values were obtained by plotting the first derivative of the fluorescence emission as a function of temperature (−dF/dT) using the cfx manager (Bio‐Rad).

## Conflicts of interest

The authors declare that they have no conflicts of interest.

## Author contributions

SS, APai, DB, GB, GPo, APao, SR, AT, GG and FC planned experiments. SS, DB, GB, RL, FRL, DC, RM, GPo, GPa, RP, AT and GG performed experiments. SS, DB, RL, GPo, APao, SR, RC, APai, AT, GG and FC analysed data. SS, DB, RL, GPo, AT, GG and FC wrote the paper. All the authors critically revised and approved the final version of the manuscript submitted for publication.

### Peer Review

The peer review history for this article is available at https://publons.com/publon/10.1111/febs.16248.
